# Structural basis for the high specificity of a *Trypanosoma congolense* immunoassay targeting glycosomal aldolase

**DOI:** 10.1371/journal.pntd.0005932

**Published:** 2017-09-15

**Authors:** Joar Pinto, Steven Odongo, Felicity Lee, Vaiva Gaspariunaite, Serge Muyldermans, Stefan Magez, Yann G.-J. Sterckx

**Affiliations:** 1 Research Unit for Cellular and Molecular Immunology (CMIM), Vrije Universiteit Brussel (VUB), Brussels, Belgium; 2 Structural Biology Research Centre, VIB, Brussels, Belgium; 3 Department of Biotechnical and Diagnostic Sciences, College of Veterinary Medicine, Animal Resources and Bio-security (COVAB), Makerere University, Kampala, Uganda; 4 Ghent Universtiy Global Campus, Yeonsu-Gu, Incheon, South Korea; Foundation for Innovative New Diagnostics (FIND), SWITZERLAND

## Abstract

**Background:**

Animal African trypanosomosis (AAT) is a neglected tropical disease which imposes a heavy burden on the livestock industry in Sub-Saharan Africa. Its causative agents are *Trypanosoma* parasites, with *T*. *congolense* and *T*. *vivax* being responsible for the majority of the cases. Recently, we identified a Nanobody (Nb474) that was employed to develop a homologous sandwich ELISA targeting *T*. *congolense* fructose-1,6-bisphosphate aldolase (*Tco*ALD). Despite the high sequence identity between trypanosomatid aldolases, the Nb474-based immunoassay is highly specific for *T*. *congolense* detection. The results presented in this paper yield insights into the molecular principles underlying the assay’s high specificity.

**Methodology/Principal findings:**

The structure of the Nb474-*Tco*ALD complex was determined via X-ray crystallography. Together with analytical gel filtration, the structure reveals that a single *Tco*ALD tetramer contains four binding sites for Nb474. Through a comparison with the crystal structures of two other trypanosomatid aldolases, *Tco*ALD residues Ala77 and Leu106 were identified as hot spots for specificity. Via ELISA and surface plasmon resonance (SPR), we demonstrate that mutation of these residues does not abolish *Tco*ALD recognition by Nb474, but does lead to a lack of detection in the Nb474-based homologous sandwich immunoassay.

**Conclusions/Significance:**

The results show that the high specificity of the Nb474-based immunoassay is not determined by the initial recognition event between Nb474 and *Tco*ALD, but rather by its homologous sandwich design. This (i) provides insights into the optimal set-up of the assay, (ii) may be of great significance for field applications as it could explain the potential detection escape of certain *T*. *congolense* strains, and (iii) may be of general interest to those developing similar assays.

## Author summary

Sub-Saharan Africa is plagued by many diseases, which impede its socio-economical development. One of these diseases, Animal African Trypanosomosis, affects livestock and is caused by the parasites of the *Trypanosoma* genus (*T*. *vivax* and *T*. *congolense*). Animal African Trypanosomosis leads to considerable economic losses and renders sustainable livestock industry in Sub-Saharan Africa very difficult. In order to proceed with the selective treatment of infected animals, they need to be properly diagnosed. We recently described the use of an assay to specifically detect *T*. *congolense* infections in both experimentally and naturally infected animals. The diagnostic assay employs a Nanobody (Nb), which is the smallest antigen-binding unit derived from camelid heavy-chain only antibodies. Our previous results showed that the Nb-based diagnostic test specifically recognizes *T*. *congolense* fructose-1,6-bisphosphate aldolase, a glycolytic enzyme that is well conserved amongst other *Trypanosoma* species. In this paper, we studied the molecular mechanisms underlying the high specificity of the Nb-based diagnostic assay. The principles derived from this work may be important for the design and improvement of similar diagnostic tests.

## Introduction

The *Trypanosoma* genus represents a diverse group of extracellular hemoflagellated parasites of which some members can infect and cause disease in humans and livestock. An infection with African trypanosomes generally leads to the development of pathologies called Human African Trypanosomosis (HAT) and Animal African Trypanosomosis (AAT), respectively. In the case of AAT (also called “Nagana”), the predominant causative agents are *T*. *congolense* and *T*. *vivax*. Estimates place 50 million animals at risk of infection in Sub-Saharan Africa and indicate that the annual AAT-driven economic losses to the local livestock industry are close to US$ 4 billion [1]. Evidently, AAT has a profound negative impact on the development of endemic regions.

While drug treatments to combat AAT exist, these are deployed in an indiscriminate fashion on a large scale due to the lack of inexpensive, specific and easy-to-use diagnostic tests. These attributes are of great significance for the development of point of care tests (POCTs) for rapid detection of AAT in a low-income setting where the disease is endemic. Importantly, the practice of indiscriminate administration of anti-trypanosomal drugs to both healthy and diseased animals has led to the emergence of drug-resistant parasite strains [2,3]. For this reason, there are ongoing efforts by the research community to develop both DNA- and protein-based tests to improve diagnosis of AAT in the field. An important category of assays for the diagnosis of trypanosomosis is the one of immunodiagnostics such as enzyme-linked immunosorbent assays (ELISAs) and lateral flow assays (LFAs) [4–9]. Both the antibody- and antigen-based immunodiagnostics have their advantages and drawbacks. The antibody-based tests, which rely on the detection of circulating parasite-induced host antibodies, have two main disadvantages: (i) a low specificity due to antibody cross-reactivity [10] and (ii) the inability of differentiating between past and ongoing infections as a consequence of long lasting circulating antibodies after parasite clearance [11–13]. These problems are alleviated by antigen-based assays, which aim to detect circulating parasite antigens. However, antigen-based diagnostic tests face their own issues. First, during an active infection, antigen levels should be high enough in order to be detected [14]. Second, the detection and capturing antibodies of the assay should bind to different epitopes from the host antibodies, which usually form immuno-complexes with the circulating antigen or at least be able to outcompete them [15]. Finally, the assay’s antibodies should be species-specific, which is not straightforward given that some antigens are highly conserved among different *Trypanosoma* species.

Recently, we described an antigen-based immunoassay for diagnosis of active *T*. *congolense* infections using Nanobody (Nb) technology [16]. The immunoassay is designed in the format of a homologous sandwich ELISA employing a single Nb (Nb474). The target of the assay was identified as *T*. *congolense* fructose-1,6-bisphosphate aldolase (*Tco*ALD) and, hence, validates this enzyme as a diagnostic biomarker for *T*. *congolense* infections [16]. Fructose-1,6-bisphosphate aldolase is an enzyme involved in the glycolytic pathway and most members of this protein family occur in solution as tetramers. This is because of the low dissociation constants for the dimer-tetramer equilibria, resulting in stable tetramer formation [17]. In trypanosomatids, most glycolytic enzymes (including aldolase) are located in specialized organelles called glycosomes [18]. At first glance, the potential of an intracellular, glycosomal enzyme as an infection biomarker may seem counterintuitive. However, it has recently been discovered that, within the context of host-parasite interactions, trypanosomes produce extracellular vesicles containing many different proteins including fructose-1,6-bisphosphate aldolase [19]. As such, *Tco*ALD is part of the *T*. *congolense* “secretome”, i.e. the collection of all molecules secreted/excreted by the parasite [20], which is probably why it can act as a biomarker for active *T*. *congolense* infections.

The Nb474-based ELISA is highly specific for *T*. *congolense* as infections with other trypanosomes such as *T*. *brucei brucei*, *T*. *vivax*, and *T*. *evansi* are not detected. Concomitantly, the Nb474 sandwich ELISA only yields a positive signal when incubated with *Tco*ALD and is negative for the detection of *Tb*ALD and *Lm*ALD (*T*. *brucei brucei* and *Leishmania mexicana* glycosomal fructose-1,6-bisphosphate aldolase) [16]. This is remarkable given that glycolytic enzymes such as fructose-1,6-bisphosphate aldolase display a relatively high degree of sequence conservation, especially among different trypanosome species (94.1% for *Tco*ALD and *Tb*ALD). These findings raise questions about the molecular details of the Nb474-*Tco*ALD interaction determining the specificity of this particular assay. In this study, we present the structural basis for the high specificity of the Nb474-based *T*. *congolense* homologous sandwich ELISA. Using a combination of X-ray crystallography, site-directed mutagenesis, ELISA and surface plasmon resonance (SPR), we demonstrate that the high specificity of the Nb474-based immunoassay is determined by its sandwich design. The results may serve as a basis for the improvement of the Nb474-based ELISA and the design of similar antigen-based diagnostic tests.

## Methods

### Cloning, protein production, and purification

The generation of Nb474 by alpaca immunization, the recombinant production of its C-terminally His-tagged variant in *E*. *coli* and subsequent purification by IMAC and size exclusion chromatography (SEC) have been described recently [16]. The recombinant production of C-terminally His-tagged *Tco*ALD^WT^ in *E*. *coli* was performed as described [16]. To purify *Tco*ALD^WT^ from an overnight production culture, cells were first harvested by centrifugation (10 min; 8000 rpm, JLA-8.1000 rotor; 14°C). The bacterial pellets were resuspended in lysis buffer (50 mM Tris-HCl, 500 mM NaCl, pH 8.0) and aliquoted in volumes of 50 ml. The aliquots were flash-frozen using liquid nitrogen and stored at -80°C. Prior to purification, aliquots were thawed on ice. Cells were lysed using a sonicator (Ultrasonic disintegrator MSE Soniprep 150; 5 sonication cycles of 1 min at 15 microns amplitude with a 2 min pause between each cycle) and the cell lysate was centrifuged (20 min, 18000 rpm, JA-20 rotor, 4°C). The supernatant was collected and filtered (0.45 μm). The purification of *Tco*ALD^WT^ was performed on an AKTA Prime Platform (GE Healthcare) using IMAC and SEC. A 5 ml HisTrap HP nickel-sepharose column (GE Healthcare) was equilibrated with buffer A (50 mM Tris-HCl, 500 mM NaCl, pH 8.0) for at least five column volumes. The sample was loaded on the column using the same buffer at a flow rate of 1 ml min^-1^. After loading, the column was further washed with 5 column volumes of the same buffer. *Tco*ALD^WT^ was then eluted by a linear gradient of buffer B (50 mM Tris-HCl, 500 mM NaCl, 1 M imidazole, pH 8.0) over 20 column volumes. The fractions containing *Tco*ALD^WT^ were pooled and concentrated to a final volume of 2 ml for the subsequent SEC step on a Superdex 200 16/60 column (GE Healthcare), which was pre-equilibrated with at least one column volume of buffer C (50 mM MES, 500 mM NaCl, pH 6.7). The sample was eluted at a flow rate of 1 ml min^-1^. Fractions containing *Tco*ALD^WT^ were pooled and stored at 4°C. Each of the purification steps was monitored by SDS-PAGE and Western blot under reducing conditions. The purification and storage conditions for *Tco*ALD^WT^ were optimized via differential scanning fluorimetry (DSF, see S2 Fig).

The *Tco*ALD^A77E^, *Tco*ALD^L106Y^, and *Tco*ALD^A77E/L106Y^ mutants were generated by modifying the *Tco*ALD^WT^ sequence. Synthesis and cloning of the mutant sequences was outsourced to a commercial company (GenScript). These mutants were produced and purified as described for *Tco*ALD^WT^.

### Generation of the Nb474-*Tco*ALD complex

The stoichiometry of the Nb474-*Tco*ALD complex was determined by analytical SEC. The experiments were performed using a Superdex 200 HR 10/30 (GE Healthcare) column, pre-equilibrated with buffer C for at least one column volume. Five hundred μl samples containing 1 mg *Tco*ALD mixed with varying molar ratios of Nb474 (Nb474:*Tco*ALD ratios of 1:4, 2:4, 3:4, 4:4, and 6:4, respectively) were allowed to incubate for at least 1 h prior to injection. The samples were eluted with a flow rate of 0.5 ml min^-1^ and the elution peaks of all chromatograms were subjected to SDS-PAGE analysis. The column was calibrated with the Bio-Rad molecular mass standard under the same conditions.

The (Nb474-*Tco*ALD)_4_ complex was generated by mixing Nb474 and *Tco*ALD in a Nb474:*Tc*oALD ratio of 6:4, allowing the sample to equilibrate for at least 1 h prior to purification on a Superdex 200 16/60 column (GE Healthcare) pre-equilibrated with at least one column volume of buffer C. The sample was eluted at a flow rate of 1 ml min^-1^. Fractions containing the (Nb474-*Tco*ALD)_4_ complex were pooled and stored at 4°C.

### Differential scanning fluorimetry

Differential scanning fluorimetry (DSF) experiments were performed to optimize the purification and storage conditions of *Tco*ALD. DSF was performed on a CFX Connect Real-Time System Thermal Cycler (Bio-Rad). Data were collected from 10°C to 95°C at a scan rate of 1°C min^-1^. The fluorescence signal was recorded every 0.5°C. Experiments were carried out in 96-well plates and the total sample volume was 25 μl. To determine the optimal protein-dye ratio, a grid screen of various concentrations of SYPRO orange dye (Life Technologies) (0x, 5x, 10x, 50x, 100x) and *Tco*ALD (0 μM, 1 μM, 5 μM, 10 μM, 25 μM, 50 μM) was carried out. After identification of a suitable condition (10x SYPRO orange dye and 5 μM of *Tco*ALD), buffer and additive screens were performed as previously described [21]. All experiments were conducted in duplicate.

### Crystallization, data collection, and data processing

The (Nb474-*Tco*ALD)_4_ complex was concentrated to 0.5 mg ml^-1^ using a 5,000 molecular weight cut-off concentrator (Sartorius Vivaspin20). Crystallization conditions were screened manually using the hanging-drop vapor-diffusion method in 48-well plates (Hampton VDX greased) with drops consisting of 2 μl protein solution and 2 μl reservoir solution equilibrated against 150 μl reservoir solution. Commercial screens from Hampton Research (Crystal Screen, Crystal Screen 2, Crystal Screen Lite, Index), Molecular Dimensions (MIDAS, JCGS+), and Jena Bioscience (JBScreen Classic 1–10) were used for initial screening. The His-tags of both proteins were retained for crystallization. The crystal plates were incubated at 20°C. Diffraction-quality crystals of the complex were obtained in Crystal Screen Lite (Hampton Research) no. 18 (100 mM sodium cacodylate pH 6.5, 200 mM magnesium acetate, 10% PEG 8000) and the crystals grew after approximately 14 days at 20°C.

The (Nb474-*Tco*ALD)_4_ crystals were cryocooled in liquid nitrogen with the addition of 25% (v/v) glycerol to the mother liquor as a cryoprotectant in 5% increments. Data were collected on the PROXIMA2 beamline at the SOLEIL synchrotron (Gif-Sur-Yvette, France) and were processed with XDS [22]. The quality of the collected data sets was verified by close inspection of the XDS output files and through phenix.xtriage in the PHENIX package [23]. Twinning tests were also performed by phenix.xtriage. Analysis of the unit-cell contents was performed with the program MATTHEWS_COEF, which is part of the CCP4 package [24]. The structure of the (Nb474-*Tco*ALD)_4_ complex was determined by molecular replacement with PHASER-MR [25] using the structure of *T*. *brucei* aldolase (PDB ID: 1F2J, [26]) as a search model. This provided a single solution (top TFZ = 96.4 and top LLG = 14236.4). From here, refinement cycles using the maximum likelihood target function cycles of phenix.refine [27] were alternated with manual building using Coot [28]. The final resolution cut-off was determined through the paired refinement strategy [29], which was performed on the PDB_REDO server [30]. The crystallographic data for the (Nb474-*Tco*ALD)_4_ complex are summarized in Table 1 and have been deposited in the PDB (PDB ID 5O0W). Molecular graphics and analyses were performed with UCSF Chimera [31].

**Table 1:** Data collection and refinement statistics. Statistics for the highest resolution shell are shown in parentheses.

	(Nb474-*Tco*ALD)_4_
**Data collection statistics**	
Wavelength (Å)	0.98
Resolution range (Å)	47.22–2.57 (2.66–2.57)
Space group	oP: P2_1_2_1_2
*a*,*b*,*c* (Å)	120.82,188.87,126.78
α,β,γ (°)	90,90,90
Mosaicity (°)	0.049
Total number of measured reflections	637286 (64553)
Unique reflections	92520 (9072)
Multiplicity	6.88 (7.11)
Completeness (%)	99.56 (99.15)
<I/σ(I)>	11.25 (1.39)
Wilson B-factor (Å^2^)	48.79
R_meas_ (%)	18.05 (149.60)
CC_1/2_ (%)	99.50 (49.80)
A.U. contains	One (Nb474-*Tco*ALD)_4_ complex
**Refinement statistics**	
CC*	0.999 (0.815)
CC_work_	0.950 (0.677)
CC_free_	0.935 (0.604)
R_work_ (%)	19.23 (32.46)
R_free_ (%)	22.21 (36.02)
Number of non-hydrogen atoms	15504
macromolecules	14952
ligands	36
solvent	516
Protein residues	1985
RMS bond lengths (Å)	0.004
RMS bond angles (°)	0.650
Ramachandran favored (%)	97.87
Ramachandran allowed (%)	1.93
Ramachandran outliers (%)	0.20
Rotamer outliers (%)	0.33
Clashscore	4.11
Overall MolProbity score	1.23
Average B-factor (Å^2^)	51.89
macromolecules	51.93
ligands	61.82
solvent	49.85
PDB ID	5O0W

### Sequence alignments and genotyping experiments

The amino acid sequences of trypanosomatid homologs of *Tco*ALD were obtained by a protein BLAST search of the TriTrypDB [32] using *Tco*ALD (Uniprot ID: G0UWE7) as a query sequence. A total of 24 trypanosomatid aldolase sequences (including *Tco*ALD) were employed to generate a sequence alignment with MAFFT [33] using the Geneious Pro program suite (Biomatters Ltd).

The amino acid sequences of *Tco*ALD homologs from *T*. *congolense* MOSROM_ALL, *T*. *congolense* SA268, *T*. *congolense* KAPEYA357, and *T*. *congolense* ZER-AGRIUMBE were kindly provided by dr. Hideo Imamura. The details on these sequences and how they were obtained have recently been published [34]. Genomic DNA samples from *T*. *congolense* TSW103, *T*. *congolense* WG84, *T*. *simiae* Ban7, and *T*. *godfreyi* Ken7 were kindly provided by Prof. dr. Wendy Gibson. More information concerning these sequences can be found in the work by Masiga *et al*. [35]. The gene encoding aldolase was extracted from these genomic DNA samples via PCR. Four different primers were designed to amplify the aldolase-coding genes based on the nucleotide sequence of the *T*. *congolense* IL3000 aldolase gene (Genbank accession number CCC93713.1): one set of primers to amplify the entire gene (*Tco*ALDcFwd: 5’-ATGTCCAGGCGTGTGGAAGTTC-3’; *Tco*ALDcRev: 5'-CTAGTAGGTGTTGCCAGCAAC-3'), a short region from the gene encoding Met1 to L181 (*Tco*ALDcFwd: 5’-ATGTCCAGGCGTGTGGAAGTTC-3’; TcoALDOcshRev: 5'-CGAGCGTTTCAGCGTTGAA-3'), or a short region from the gene encoding Y162 to Y372 (*Tco*ALDcshFwd: 5-ACAAGATTCAGAACGGCAC-3'; *Tco*ALDcRev: 5'-CTAGTAGGTGTTGCCAGCAAC-3'). The PCR mix contained the following components: 0.4 mM forward primer, 0.4 mM reverse primer, 0.4 mM dNTPs, 1x GoTaq G2 buffer (Promega), 1.5 U GoTaq G2 DNA polymerase (Promega), 5 ng genomic DNA. The PCR was performed according to the following protocol: (i) 30 cycles of denaturation (95°C, 5 min), denaturation (94°C, 1 min), annealing (55°C, 1.5 min), elongation (72°C, 1 min), (ii) elongation (72°C, 10 min), (iii) storage (4°C). Amplified PCR products were resolved by electrophoresis on 1% agarose (Lonza) in TBE buffer (90 mM Tris, 90 mM borate, 2.5 mM EDTA). Electrophoresis was conducted at 100V for 30 minutes. Amplicons were cleaned-up using the PCR clean-up kit (Sigma-Aldrich) following the protocol recommended by the manufacturer. Gene sequences were obtained through DNA sequencing with 50 pmol of each primer. Sequencing of the samples was outsourced to the VIB Genetic Service Facility. A total of 16 aldolase sequences were employed to generate a sequence alignment with MAFFT [33] using the Geneious Pro program suite (Biomatters Ltd).

### ELISAs

The Nb474H/Nb474B homologous and Nb474B/mouse anti-His heterologous sandwich ELISAs were performed in similar manner as previously described [16]. Briefly, Nb474H (homologous ELISA) or Nb474B (heterologous ELISA) was coated on the plate as capture reagent by applying 100 μl (diluted to a concentration of 0.02 μg ml^-1^ in PBS) per well. The plate was incubated overnight at 4°C and the excess of non-coated Nb was removed by washing the plate three times with PBS containing 0.01% Tween20 (PBS-T). Next, blocking of residual protein binding sites was performed by adding 300 μl blocking buffer (5% milk powder in PBS) to each well and the plate was kept for 2 h at room temperature. Subsequently, the plate was washed three times with PBS-T, after which the *Tco*ALD wild type and mutant variants were allowed to interact with the coated Nb by applying 100 μl (diluted to a concentration of 1 μg ml^-1^ in blocking buffer) per well. After incubation for 1 h at room temperature, the plates were subsequently washed three times with PBS-T. Then, 100 μl Nb474B (diluted to a concentration of 0.02 μg ml^-1^ in blocking buffer) or 100 μl mouse anti-His (diluted to a concentration of 0.05 μg ml^-1^ in blocking buffer) was added to the plate as a primary detection reagent for the homologous and heterologous ELISAs, respectively. After an incubation of 1 h at room temperature, the plate was washed 5 times with PBS-T. The conjugate, 100 μl of streptavidin-HRP (diluted to a concentration of 1 μg ml^-1^ in rinsing buffer) or 100 μl goat anti-mouse-HRP (diluted to a concentration of 0.05 μg ml^-1^ in blocking buffer), was then added to the plate for the homologous and heterologous ELISAs, respectively, followed by incubation for 1 h at room temperature. After a final washing step (5 times with PBS-T), the ELISAs were developed by addition of 100 μl of 3,3’,5,5’-tetramethylbenzine (TMB) substrate and subsequent incubation for 25 min at room temperature. The enzymatic reaction was stopped by adding 50 μl 1 M H_2_SO_4_ to the reaction mixture. The plates were read at OD_450 nm_ with a VersaMax ELISA Microplate Reader (Molecular Devices).

### Surface plasmon resonance

Surface plasmon resonance (SPR) experiments were performed on a BIAcore T200 system (GE Healthcare). The interactions between Nb474 and the *Tco*ALD variants were analyzed on a CM5 chip. Nb474 was immobilized in flow cell 2 using the following procedure. Using a flow rate of 5 μl min^-1^ the carboxylated dextran matrix was activated by a 7-min injection of a solution containing 0.2 M N-ethyl-N′-(3-diethylamino)propyl carbodiimide (EDC) and 0.05 M N-hydroxysuccinimide (NHS). A Nb474 solution of 1 μg ml^-1^ (50 mM sodium acetate pH 5.0) was subsequently injected until the desired amount of protein was immobilized (approx. 50 R.U.). The surface immobilization was then blocked by a 7-min injection of 1 M ethanolamine hydrochloride. The surface in flow cell 1 was used as a reference and treated only with EDC, NHS and ethanolamine. Sensorgrams for different concentrations of the *Tco*ALD variants expressed as monomer concentrations (0.02 nM, 0.05 nM, 0.10 nM, 0.20 nM, 0.35 nM, 0.50 nM, 0.75 nM, 1.00 nM, 2.00 nM, 5.00 nM, 10.00 nM for *Tco*ALD^WT^; 0.78 nM, 1.56 nM, 3.12 nM, 6.25 nM, 12.50 nM, 25.00 nM, 50.00 nM, 100.00 nM, 125.00 nM, 250.00 nM, 500.00 nM for *Tco*ALD^A77E^; 0.12 nM, 0.24 nM, 0.49 nM, 0.98 nM, 1.95 nM, 3.90 nM, 7.81 nM, 15.62 nM, 31.25 nM, 62.50 nM, 125.00 nM for *Tco*ALD^L106Y^; 0.78 nM, 1.56 nM, 3.12 nM, 6.25 nM, 12.50 nM, 25.00 nM, 50.00 nM, 100.00 nM, 125.00 nM, 250.00 nM, 500.00 nM, 750.00 nM, 1.00 μM for *Tco*ALD^A77E/L106Y^) plus a 0 concentration (injection of running buffer) were collected at a flow rate of 30 μl min^-1^ and a data collection rate of 1 Hz.

For the Nb474 binding/washing experiments, the ligand (Nb474) was first saturated by an injection of an adequate concentration of the first analyte (*Tco*ALD variant; 10 nM for *Tco*ALD^WT^, 125 nM for *Tco*ALD^A77E^, 31.25 nM for *Tco*ALD^L106Y^, and 750 nM for *Tco*ALD^A77E/L106Y^). Immediately after injection of the first analyte (i.e., no dissociation phase), different concentrations of the second analyte (Nb474; 0.5 nM, 1.0 nM, 1.5 nM, 5.0 nM, 10.0 nM, 100.0 nM, 500.0 nM, 1.0 μM) plus a 0 concentration (injection of running buffer) were injected at a flow rate of 30 μl min^-1^ and a data collection rate of 1 Hz.

All analytes were dialyzed into the running buffer (20 mM HEPES, 150 mM NaCl, 0.005% Tween, 3.4 mM EDTA, pH 7.4) prior to data collection. Analyte injections were performed with association and dissociation phases of 480 s and 660 s, respectively. This was followed by a 5 μl pulse injection of regeneration buffer (0.2% SDS). Prior to data analysis, reference and zero concentration data were subtracted from the sensorgrams. The data collected for Nb474 binding to the pre-formed Nb474-*Tco*ALD^WT^ and Nb474-*Tco*ALD^A77E^ complexes were analyzed with a 1:1 Langmuir binding model.

All experiments were performed on the same sensor chip using the same flow channels.

## Results

### *Tco*ALD tetramer contains four binding sites for Nb474

The amino acid sequences of glycolytic enzymes such as fructose-1,6-bisphosphate aldolase are generally well conserved. Indeed, among trypanosomatids, the pairwise sequence identity for aldolase is 86.7% (S1 Fig). For *Tco*ALD and *Tb*ALD, the sequence identity is 94.1%. Nonetheless, the Nb474-based immunoassay is highly specific for *Tco*ALD. Thus, we were interested in identifying the *Tco*ALD epitope recognized by Nb474.

**Fig 1:**
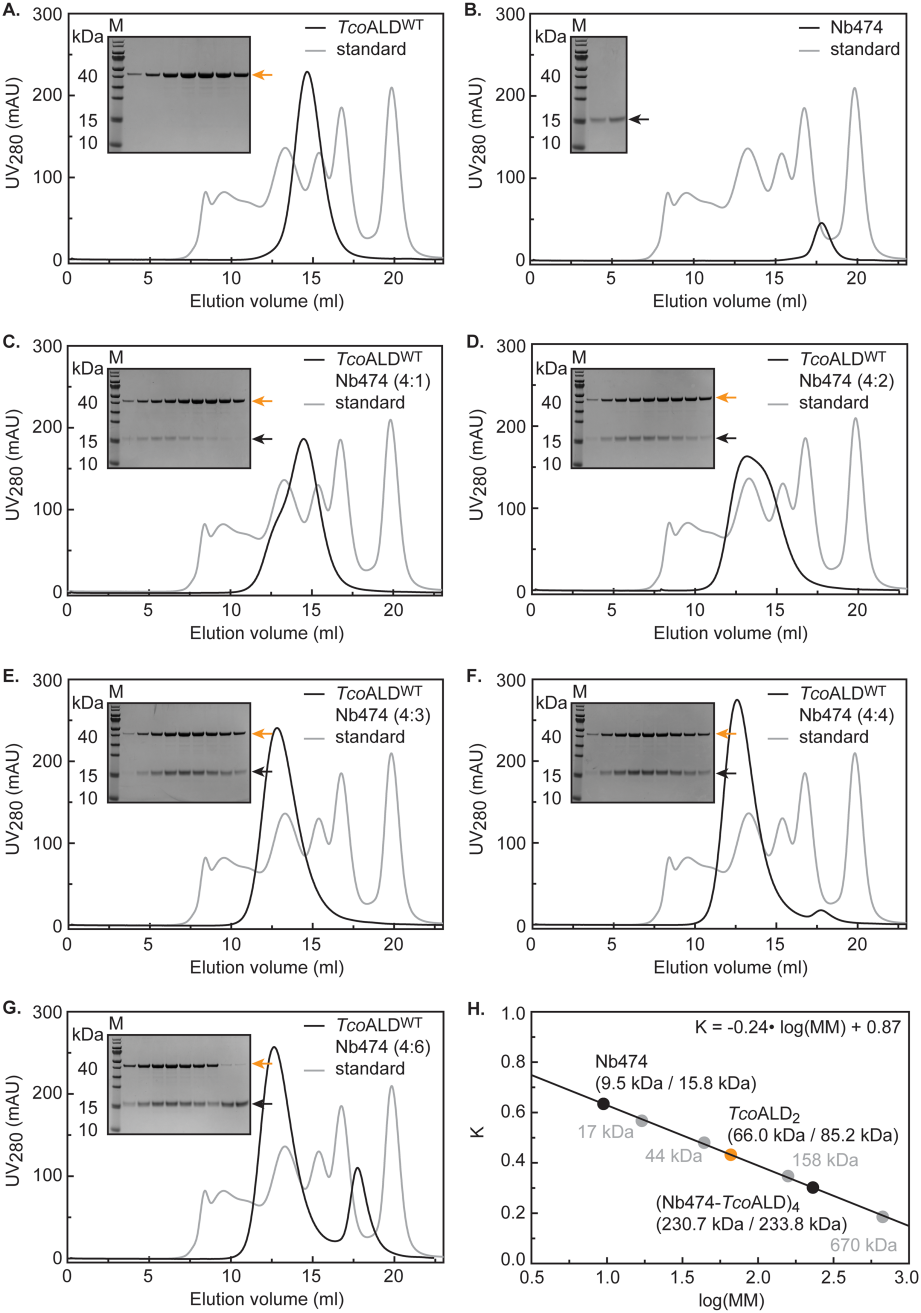
Investigation of the stoichiometry of the Nb474-*Tco*ALD complex by analytical SEC. (A.-G.) Analytical SEC on the purified *Tco*ALD (A.), Nb474 (B.) and samples containing *Tco*ALD and Nb474 mixed at different molar ratios: 4:1 (C.), 4:2 (D.), 4:3 (E.), 4:4 (F.), and 4:6 (G.). All experiments were performed on a Superdex 200 HR 10/30 column. The black and grey traces represent the chromatograms of the different protein samples and the BioRAD gel filtration standard, respectively. In all figures, the inset shows an SDS-PAGE analysis of the elution peaks. *Tco*ALD (MM = 42.6 kDa) and Nb474 (MM = 15.8 kDa) are indicated by the orange and black arrows, respectively. *Lane M*, Prestained Protein Molecular Weight Marker (Fermentas). (H.) The calibration of the Superdex 200 HR 10/30 column that allows estimation of molecular mass based on the sample’s elution volume. The values between brackets indicate the estimated molecular mass versus the theoretical mass of the sample under investigation.

First, we produced *Tco*ALD through recombinant protein production in *E*. *coli* and optimized its purification conditions via DSF. The details of these procedures are given in Materials and Methods and S2 Fig (panels A-D). Next, we determined the stoichiometry of the Nb474-*Tco*ALD complex via analytical SEC (Fig 1). An excess of Nb474 could only be detected at a molar ratio of 6:4 between Nb474 and the *Tco*ALD monomer, and not at the other tested ratios 1:4, 2:4, 3:4, and 4:4. This suggests that one Nb474 binds a single *Tco*ALD monomer. The analytical SEC reveals another interesting feature of the Nb474-*Tco*ALD interaction. First, *Tco*ALD appears to occur as a dimer in solution. The *Tco*ALD monomer has a theoretical molecular mass of 42.6 kDa (170.4 kDa for a *Tco*ALD tetramer). Instead, *Tco*ALD migrates with a higher apparent molecular mass (MM_app_ = ~66 kDa, Fig 1H), suggesting a dimer population (*Tco*ALD_2_). Second, a comparison of the analytical SEC profiles recorded for the different ratios between Nb474 and the *Tco*ALD monomer suggests that adding Nb474 to *Tco*ALD promotes tetramer formation. Rather than shifting the *Tco*ALD_2_ peak to the left, the titration of Nb474 reduces the intensity of the *Tco*ALD_2_ peak and gives rise to a peak corresponding to an entity of larger molecular mass. At an estimated molecular mass of ~230 kDa for the peak at a 4:4 molar ratio (Fig 1F), this complex likely corresponds to a hetero-octameric (Nb474-*Tco*ALD)_4_ complex in which four Nb474 occupy identical sites on the *Tco*ALD tetramer (*Tco*ALD_4_). Indeed, the theoretical molecular mass of such a complex is 233.52 kDa, which is in accordance with the molecular mass calculated based on the analytical SEC data (Fig 1H).

**Fig 2:**
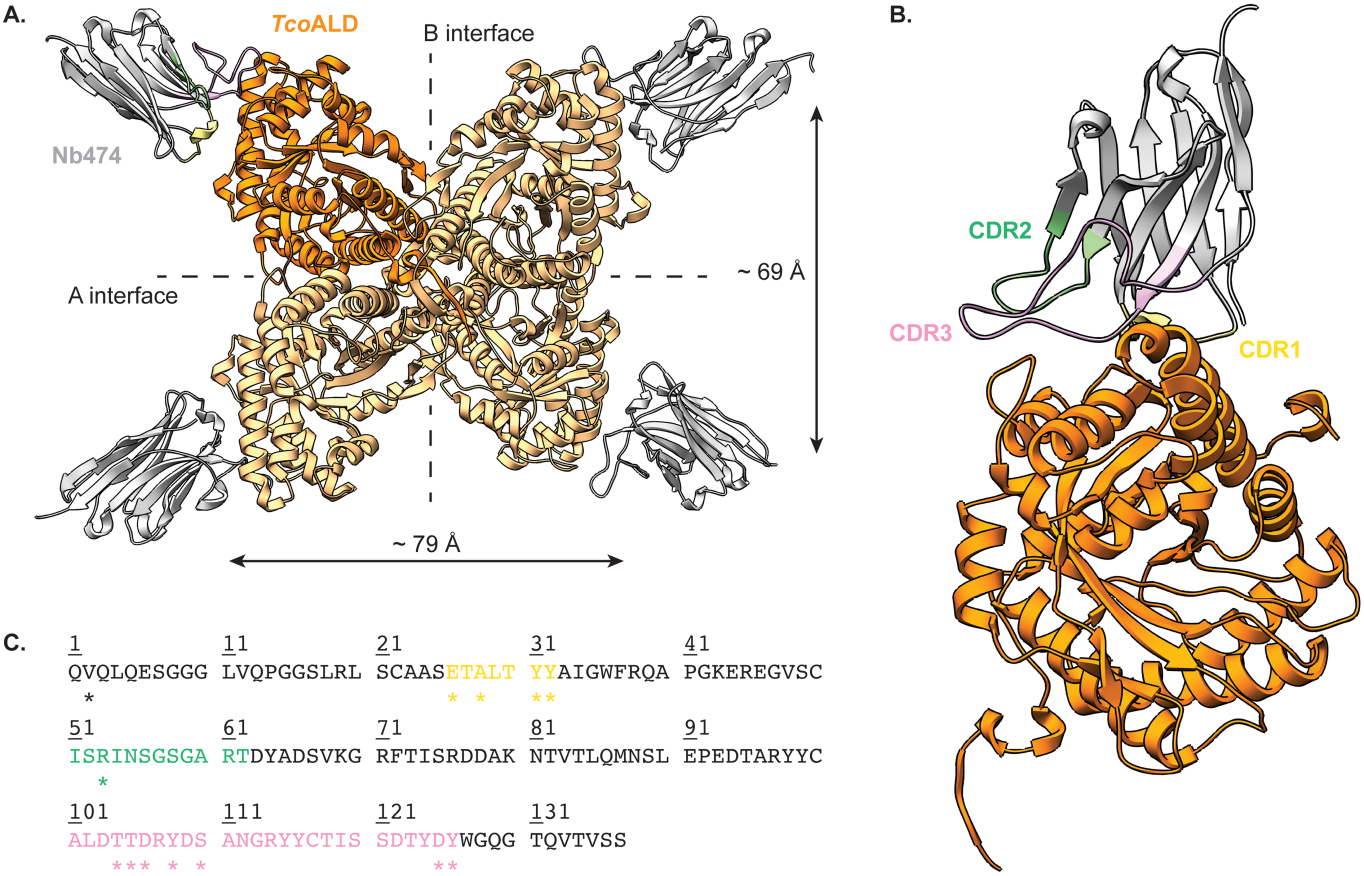
Crystal structure of the (Nb474-*Tco*ALD)_4_ complex. (A.) Overview of the entire complex. The *Tco*ALD tetramer and Nb474 are colored in orange and grey, respectively. The A and B dimer interfaces of *Tco*ALD are indicated, together with the distances between the Nb474 epitopes relative to these interfaces. (B.) Close-up of the interaction between Nb474 and a *Tco*ALD monomer. Color codes are as in (A.) and the Nb474 CDRs are indicated in different colors (CDR1, yellow; CDR2, green; CDR3, pink). (C.) Amino acid sequence of Nb474. The CDRs are highlighted in the same colors as in (B.). The residues marked by the asterisk ‘*’ are part of the Nb474 paratope and are involved in epitope binding. More details given in S3 Fig and S1 Table.

For crystallization purposes, the (Nb474-*Tco*ALD)_4_ complex was prepared using a 6:4 molar ratio as described above and purified by SEC. Crystals of the (Nb474-*Tco*ALD)_4_ complex and their diffraction are shown in S2 Fig (panels E-F). The details of the crystallographic experiment are summarized in Table 1. The crystal structure of the (Nb474-*Tco*ALD)_4_ complex confirms that *Tco*ALD_4_ indeed contains 4 binding sites for Nb474 (Fig 2). Nb474 binds an epitope on the *Tco*ALD surface that is located relatively far away from the aldolase A and B dimer interfaces. This results in large distances between the Nb474 epitopes on TcoALD_4_ relative to the A and B dimer interfaces (~ 69 Å and ~ 79 Å from one epitope to another, respectively). The Nb474-*Tco*ALD interaction is mediated by residues from all three complementarity determining regions (CDRs; Fig 2). The bulk of the contacts are provided by CDR1 and CDR3, while a single amino acid from CDR2 (Arg53) is involved in *Tco*ALD recognition. A detailed overview of all the interactions is given in S3 Fig and S1 Table.

### Comparing the *Tco*ALD epitope recognized by Nb474 to corresponding regions of other trypanosomatid aldolases

**Fig 3:**
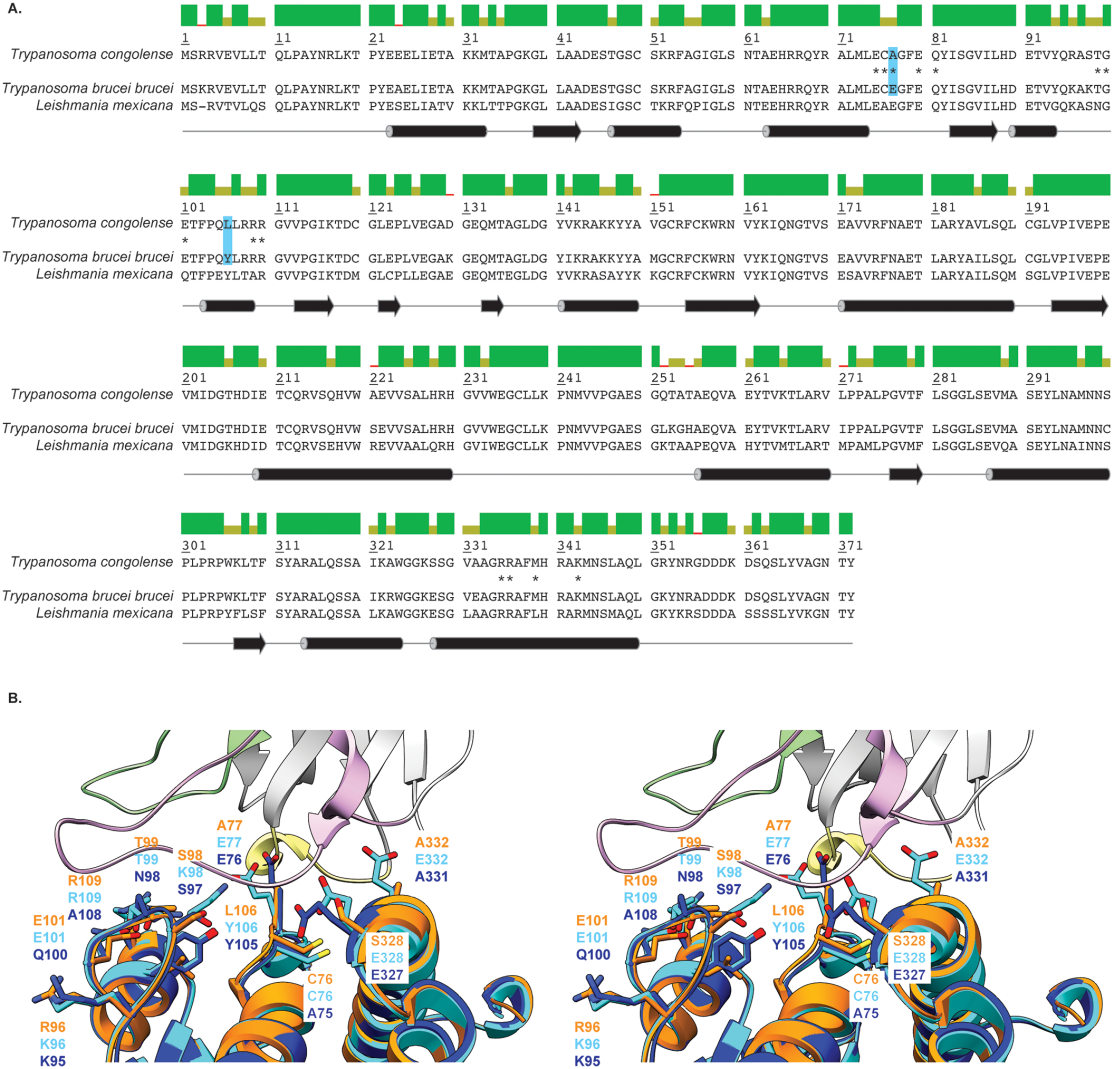
Comparison of *Tco*ALD to other trypanosomatid aldolases. (A.) Sequence alignment between *Tco*ALD, *Tb*ALD, and *Lm*ALD. The colored bars above the sequence alignment represent the percentage of sequence identity: green (100%), green-brown (between 30% and 100%), and red (below 30%). The corresponding secondary structure elements are shown below the sequence alignment. Cylinders and arrows represent α-helices and β-sheets, respectively. The *Tco*ALD residues involved in the interaction with Nb474 are marked by an asterisk ‘*’. The residues highlighted in blue were selected for site-specific mutagenesis. (B.) Stereo view of a superposition of the crystal structures of the Nb474-*Tco*ALD complex (this work, color code as in Fig 2), *Tb*ALD (PDB ID: 1F2J, [26], depicted in cyan), and *Lm*ALD (PDB ID: 1EPX, [26], depicted in blue). Those residues that are located in the vicinity of or on the *Tco*ALD epitope recognized by Nb474 and are distinct between the three trypanosomatid aldolases are shown in stick representation.

A superposition of the crystal structures of *Tb*ALD (PDB ID: 1F2J, [26]), *Lm*ALD (PDB ID: 1EPX, [26]), and the (Nb474-*Tco*ALD)_4_ complex allows to pinpoint those residues that are located in the vicinity of or on the *Tco*ALD epitope recognized by Nb474 and are distinct between the three trypanosomatid aldolases (Fig 3A). These residues are located at positions 76, 77, 96, 98, 99, 101, 109, 328, and 332 for *Tco*ALD and *Tb*ALD. For *Lm*ALD, all positions are shifted by -1.

We reasoned that mutating some of these *Tco*ALD residues to their *Tb*ALD/LmALD counterparts would influence Nb474 binding and provide a starting point to explain the assay’s specificity. Within the above-mentioned selection of amino acids, we first identified those residues that are conserved in both *Tb*ALD and *Lm*ALD, but differ in *Tco*ALD. These amino acids would most likely contribute to a loss of binding energy given that the Nb474-based immunoassay does not provide a binding signal for both *Tb*ALD and *Lm*ALD [16]. This narrowed the selection of residues to mutate down to four positions: A77/E77, R96/K96, L106/Y106, and S328/E328 for *Tco*ALD/*Tb*ALD (E76, K95, Y105, and E327 for *Lm*ALD). Positions 96 and 328 were further omitted because, based on the structural comparison depicted in Fig 3B, these are too far from the Nb474 paratope to have any influence on Nb474-*Tco*ALD interaction. This resulted in a final selection of two positions that were targeted for site-specific mutagenesis: A77/E77 and L106/Y106 for *Tco*ALD/*Tb*ALD (E76 and Y105 for *Lm*ALD). Hence, three *Tco*ALD mutants (*Tco*ALD^A77E^, *Tco*ALD^L106Y^, and *Tco*ALD^A77E/L106Y^) were generated.

### The high specificity of the Nb474-based immunoassay is determined by its sandwich design

**Fig 4:**
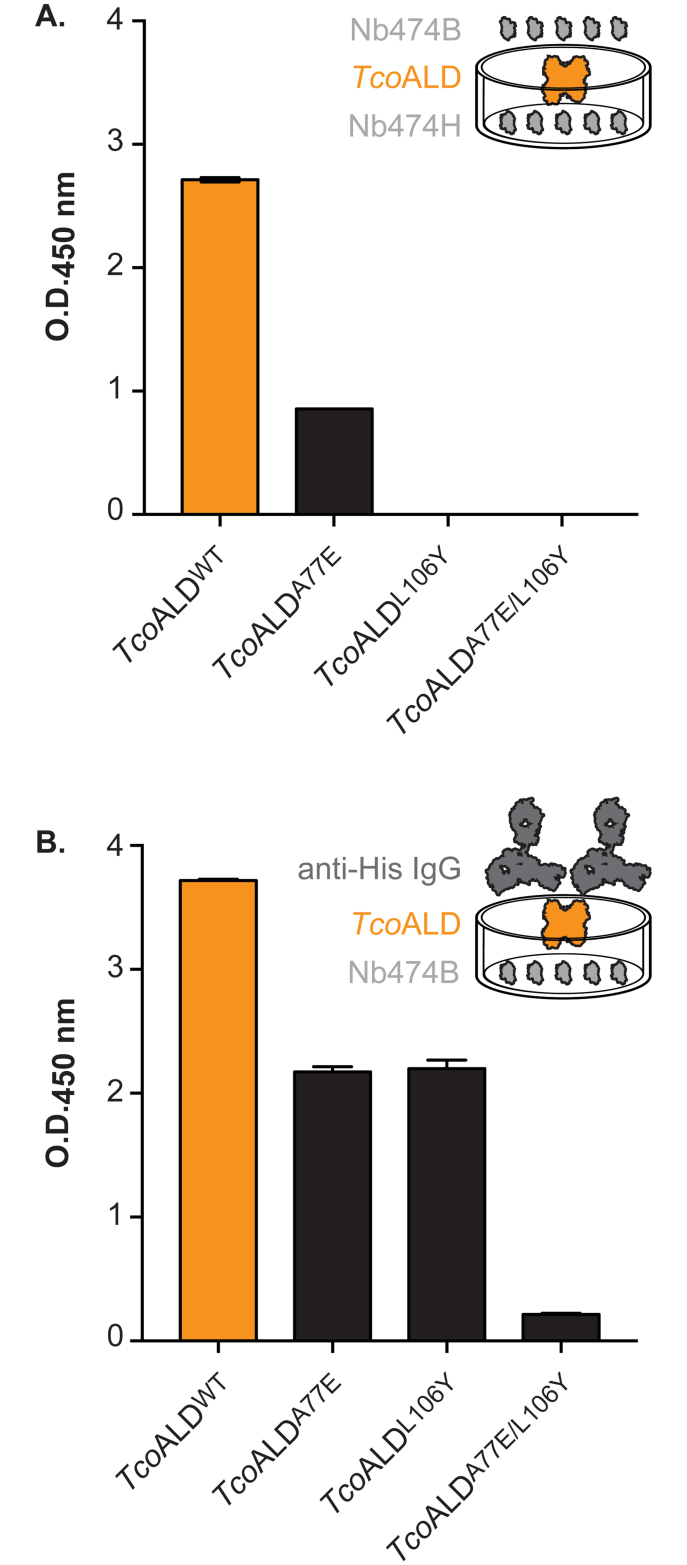
Homologous and heterologous sandwich ELISAs on the *Tco*ALD variants. (A.) Homologous sandwich ELISA on the *Tco*ALD variants in which His-tagged (Nb474H) and biotinylated (Nb474B) Nb474 were employed as capturing and detecting antibodies, respectively. (B.) Heterologous sandwich ELISA on the *Tco*ALD variants in which biotinylated Nb474 (Nb474B) and mouse anti-His IgG were employed as capturing and detecting antibodies, respectively.

The different *Tco*ALD variants were tested in the Nb474-based homologous sandwich ELISA and compared. As can be seen from Fig 4A, *Tco*ALD^A77E^ is still detected, although to a lesser extent compared to *Tco*ALD^WT^, whereas *Tco*ALD^L106Y^ and *Tco*ALD^A77E/L106Y^ display no signal. Two hypotheses could be presented to explain these observations. The first poses that the lack of detection of the *Tco*ALD mutants is caused by a loss of recognition by Nb474 due to the introduced mutations. The second explanation states that the mutations somehow weaken the Nb474-*Tco*ALD interaction and that a “self-competition” or “washing” effect is at play because of the homologous sandwich design of the assay. In order to distinguish between both hypotheses, a second, heterologous ELISA was carried out with Nb474B as a capturing agent (Fig 4B). Compared to *Tco*ALD^WT^, the three mutants display a lower but clear signal, with *Tco*ALD^A77E/L106Y^ providing the lowest intensity. When combined, the results of both ELISAs suggest that Nb474 still interacts with the *Tco*ALD mutants, thus favoring the second hypothesis.

**Fig 5:**
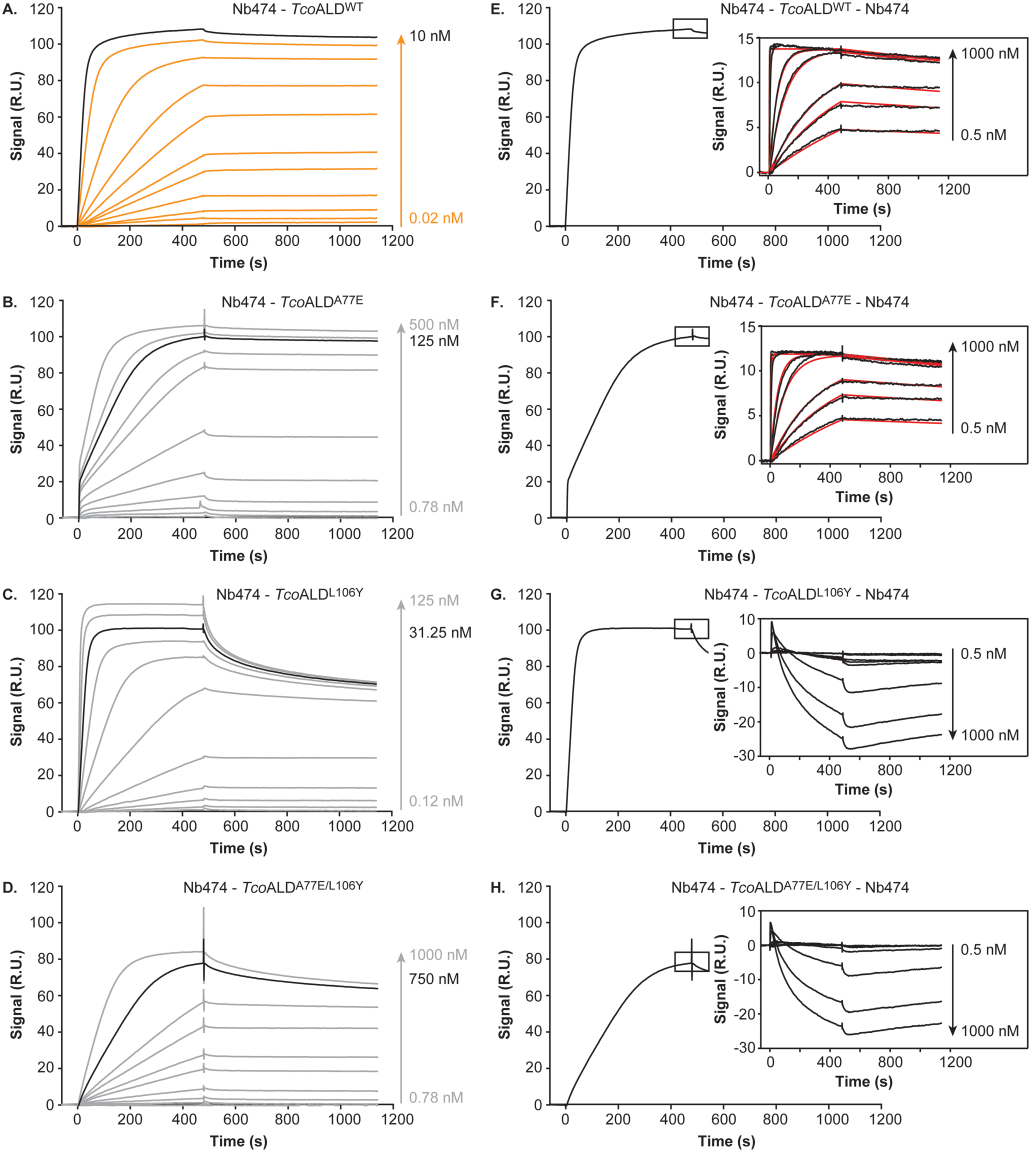
Investigation of the interaction between Nb474 and the *Tco*ALD variants by SPR. (A.-D.) SPR data recorded for the Nb474-*Tco*ALD^WT^ (A.), Nb474-*Tco*ALD^A77E^ (B.), Nb474-*Tco*ALD^L106Y^ (C.), and Nb474-*Tco*ALD^A77E/L106Y^ (D.) interactions with Nb474 as ligand and the *Tco*ALD variants as analytes. For *Tco*ALD^WT^ the sensorgrams are shown in orange, while the experimental traces are displayed in grey for the *Tco*ALD mutants. In all panels, the sensorgram in black indicates the concentration of analyte used to saturate the Nb474-coated sensor chip for the SPR sandwich assay. (E.-H.) Results of the SPR sandwich assay for the Nb474-*Tco*ALD^WT^-Nb474 (E.), Nb474-*Tco*ALD^A77E^-Nb474 (F.), Nb474-*Tco*ALD^L106Y^-Nb474 (G.), and Nb474-*Tco*ALD^A77E/L106Y^-Nb474 (H.) interactions with Nb474 as ligand and the *Tco*ALD variants as analytes. Upon saturation of the Nb474-coated sensor surface with analyte (saturation obtained after ~500 s and indicated by the black rectangle), varying concentrations of Nb474 are injected onto the sensor chip surface. In all panels, the resulting sensorgrams (black traces) are shown in the insets. For the Nb474-*Tco*ALD^WT^-Nb474 (E.) and Nb474-*Tco*ALD^A77E^-Nb474 (F.) sandwiches the additional binding of Nb474 was fitted with a 1:1 Langmuir binding model (red traces). The concentrations for all *Tco*ALD variants are expressed in terms of monomer concentrations.

The interaction between Nb474 and each of the *Tco*ALD variants was investigated further via SPR. For this experiment, Nb474 and the *Tco*ALD variants were employed as ligand and analytes, respectively. From Fig 5 (panels A-D), it is clear that all *Tco*ALD mutants bind to Nb474 and that the kinetics of the Nb474-*Tco*ALD interaction are affected by the A77E, L106Y, and A77E/L106Y mutations. Unfortunately, this could not be quantified by any interaction model, which is why the interpretation of the presented SPR data is performed in a semi-quantitative fashion. For *Tco*ALD^WT^, saturation is readily observed at an enzyme concentration of 10 nM (maximal binding signal R_max_ of ~110 R.U.; Fig 5A). This indicates that the affinity of the Nb474-*Tco*ALD^WT^ interaction is quite high (nM to pM range), which is supported by the necessity of solutions containing 0.2% SDS to regenerate the Nb474-coated sensor chip surface. The three *Tco*ALD mutants only attain a similar maximal binding signal at higher analyte concentrations (Fig 5, compare panels A-D), suggesting that the binding of Nb474 to the *Tco*ALD mutants is weakened by the introduced mutations. Interestingly, although *Tco*ALD^A77E^ only reaches a binding signal of ~110 R.U. at a concentration of 500 nM (Fig 5B), the dissociation phases for the Nb474-*Tco*ALD^WT^ and Nb474-*Tco*ALD^A77E^ interactions seem very similar. In the case of *Tco*ALD^L106Y^, a signal of ~110 R.U. is attained at a concentration of 125 nM (Fig 5C), while the dissociation of this complex appears to occur faster. Finally, binding of Nb474 to *Tco*ALD^A77E/L106Y^ does not reach the maximal signal observed for the other *Tco*ALD variants, even at a concentration of 1 μM (Fig 5D).

An additional SPR experiment was designed to mimic the homologous sandwich ELISA (Fig 5, panels E-H). The basic set-up is the same as mentioned above: Nb474 and the *Tco*ALD variants were selected as ligand and analytes, respectively. For each *Tco*ALD variants, the analyte concentration was chosen to saturate the Nb474-coated sensor chip surface. Upon saturation, Nb474 was injected onto the sensor chip surface at different concentrations and allowed to interact with the pre-formed Nb474-*Tco*ALD complex. For both *Tco*ALD^WT^ and *Tco*ALD^A77E^, this results in additional binding of Nb474 and formation of Nb474-*Tco*ALD^WT^-Nb474 and Nb474-*Tco*ALD^A77E^-Nb474 sandwiches (Fig 5, panels E and F). Interestingly, these binding curves can be fitted with a 1:1 Langmuir binding model. It appears that the Nb474-*Tco*ALD interaction is virtually unaffected by the A77E mutation as the affinity constants for both binding events are quasi identical (Table 2). In the case of *Tco*ALD^L106Y^ and *Tco*ALD^A77E/L106Y^, the injection of additional Nb474 leads to dissociation of the pre-formed Nb474-*Tco*ALD complex as evidenced by a reduction in RU signal over time (Fig 5, panels G and H).

**Table 2:** Kinetic parameters of the Nb474-*Tco*ALD^WT^ and Nb474-*Tco*ALD^A77E^ interactions.

	k_on_ (M^-1^ s^-1^)	k_off_ (s^-1^)	K_D_ (pM)	χ^2^
**Nb474-*Tco*ALD^WT^**	1.87 10^6^	1.38 10^−4^	73.83	0.05
**Nb474-*Tco*ALD^A77E^**	2.11 10^6^	1.41 10^−4^	66.97	0.05

The ELISA data in conjuncture with the SPR results indicate that the high specificity for *Tco*ALD displayed by the Nb474-based immunoassay is not determined by the initial interaction between *Tco*ALD and the capturing Nb474, but rather from its homologous sandwich design.

### Aldolase sequence conservation in the *Nannomonas* subgenus

The above-mentioned mutation studies imply that *T*. *congolense* strains carrying mutations at positions 77 and/or 106 would be detected less efficiently (or not at all) by the Nb474-based homologous sandwich ELISA.

To probe the aldolase sequence variation within the *Trypanosoma* subgenus *Nannomonas*, of which *T*. *congolense* is a member, the aldolase amino acid sequences were determined for the following parasites: *T*. *congolense* (Savannah type), *T*. *congolense* (Forest type), *T*. *congolense* (Kilifi type), *T*. *simiae*, and *T*. *godfreyi*. A sequence alignment reveals that the sequence identity of aldolase within *Nannomonas* is relatively high (90.6%; S4 Fig). Interestingly, while position 106 remains unaltered throughout all sequenced *Nannomonas* members (Leu106), position 77 displays a larger sequence variation: *T*. *congolense* Savannah and Kilifi subtypes contain an Ala77, *T*. *congolense* Forest subtypes harbor a Val77, and *T*. *simiae* and *T*. *godfreyi* both possess a Glu residue at position 77 (S4 Fig).

## Discussion

Animal African Trypanosomosis is neglected on a global scale, yet it continues to impose a heavy societal and economic burden on Sub-Saharan Africa [1]. However, the disease can be contained provided that the necessary control and surveillance programs are put in place. For HAT, such multidisciplinary initiatives have “*eliminated the disease as a public health problem*” [36], which means that in most areas HAT can be targeted for eradication. Together with vector control strategies and adequate treatment schemes, tools for rapid diagnosis of AAT are of utmost importance. Luckily, such assays targeting *T*. *congolense* and *T*. *vivax* infections (the two important causative agents of AAT in livestock) are under development [4–7,9]. Recently, we described the generation of a highly specific Nb-based homologous sandwich ELISA targeting *Tco*ALD to detect active *T*. *congolense* infections [16].

Evidently, the principle of a homologous sandwich ELISA can only work if the target antigen is a multimer [37,38]. Members of the fructose-1,6-bisphosphate aldolase family usually occur in solution as stable tetramers [17]. Mutations at the A and B dimer interfaces influence the dimer-tetramer equilibria by destabilizing the tetramer, but, interestingly, without affecting the enzyme’s catalytic activity [17,39,40]. While aldolase dimers retain the same catalytic potential compared to tetramers, they appear to be less thermostable [39]. In the case of *Tco*ALD, the analytical SEC data presented here indicates that the enzyme does not occur as a tetramer in solution, but rather seems to behave as a dimer. This suggests that the dissociation constants for the dimer-tetramer equilibria are higher for *Tco*ALD compared to archetypal aldolases. This may also explain why *Tco*ALD was observed to be labile during our first purification trials. Using DSF, we markedly improved the thermal stability of *Tco*ALD (melting temperatures T_m_ of ~40°C and ~50°C in the initial and final buffer conditions, respectively). Interestingly, in their work on rabbit muscle aldolase, Beernink and Tolan measured T_m_ values of ~45°C and ~60°C for aldolase dimers and tetramers, respectively [39]. Nb474 clearly influences the *Tco*ALD dimer-tetramer equilibria. As shown by analytical SEC, the titration of Nb474 against TcoALD shifts the equilibrium towards the formation of an aldolase tetramer. The end-point of the titration is reached at a Nb474:*Tco*ALD molar ratio of 4:4, suggesting the formation of a hetero-octameric (Nb474-*Tco*ALD)_4_ complex, which is confirmed by X-ray crystallography.

The crystal structure of the (Nb474-*Tco*ALD)_4_ complex provides a molecular basis as to why the homologous sandwich ELISA format works in the case of *Tco*ALD. The Nb474 epitope is located on the extremities of the *Tco*ALD tetramer, thereby easily allowing all four copies of Nb474 to bind their epitopes without mutual interference. A detailed analysis of the Nb474-*Tco*ALD interface reveals a multitude of interactions between both proteins, mainly mediated by CDR1 and CDR3 residues. The SPR data demonstrate that these interactions result in a high-affinity recognition event (K_D_ in the pM range), which explains why Nb474 is such a good capturing agent [16]. In some cases, the mutation of a single residue on the antigen’s epitope can cause total loss of antigen recognition by the Nb [41]. In contrast, the mutation studies presented here indicate that this is not the case for the Nb474-*Tco*ALD interaction. Changing specific *Tco*ALD epitope residues to their *Tb*ALD/*Lm*ALD counterparts (A77E, L106Y, A77E/L106Y) does not result in a loss of *Tco*ALD recognition by Nb474. Instead, an interaction still takes place, albeit with different kinetics, suggesting that the mutations have a significant effect on *Tco*ALD binding. Unfortunately, this could not be quantified by any interaction model. This indicates that the interactions taking place on the sensor chip surface are relatively complex. Indeed, based on the analytical SEC results, we suspect that multiple events occur simultaneously on the sensor chip surface. First, *Tco*ALD occurs as a dimer in solution making it a bivalent analyte. Hence, this possibly leads to avidity effects on the sensor chip, whereby one *Tco*ALD_2_ is able to bind two Nbs simultaneously. The use of an analysis model designed to take such effects into account was attempted [42], but this did not improve the fit. Second, since Nb474 binding promotes *Tco*ALD_2_ tetramer formation, this would mean that, on the sensor chip surface, binding of a *Tco*ALD_2_ to a Nb allows the subsequent recruitment of an additional *Tco*ALD_2_ onto a formed Nb474-*Tco*ALD_2_ complex. Moreover, the effect of the introduced mutations on the *Tco*ALD dimer-tetramer equilibria is unknown. Generally, in such complex cases, the ‘analyte’ should be immobilized on the sensor surface to become ‘ligand’ and the ‘ligand’ should be used in the mobile phase to become ‘analyte’. However, employing the *Tco*ALD variants as ligands is not an option as they do not survive the harsh regeneration condition used during the experiment (0.2% SDS). Given the complexity of the interactions on the sensor chip surface, we therefore prefer not to fit the data with any model to avoid overparametrization and misinterpretation of the real K_D_ value describing the Nb474-*Tco*ALD interaction. Hence, we interpreted the SPR data in a semi-quantitative manner.

A determination of the dissociation affinity constants for the Nb474-*Tco*ALD^WT^ and Nb474-*Tco*ALD^A77E^ becomes possible when the SPR experiments are carried out according to the format of the homologous sandwich ELISA. Surprisingly, both interactions have very similar affinities (73.83 pM and 66.97 pM, respectively) despite the mutation of an Ala to a bulkier, charged Glu residue. This can be explained by a closer examination of the Nb474 paratope (S5A Fig). Nb474 contains a cavity, which is perfectly aligned with the position of Ala77 on the *Tco*ALD epitope. Hence, given a local rearrangement, a Glu side chain could be easily accommodated. We hypothesize that Nb474 immobilized in an ELISA well or on a sensor chip surface has less conformational freedom to accommodate the Glu77 side chain, which leads to less efficient binding of *Tco*ALD^A77E^. This explains why, during the SPR experiments, a 50-fold increase in analyte concentration was needed for *Tco*ALD^A77E^ compared to *Tco*ALD^AWT^ in order to reach the same binding signal. However, once bound, the Nb474-*Tco*ALD^A77E^ dissociation displays the same kinetics as for the Nb474-*Tco*ALD^WT^ interaction as evidence by the SPR data. In contrast, non-immobilized Nb474 has the conformational freedom to accommodate Glu77 on *Tco*ALD^A77E^ efficiently, thereby displaying very similar binding kinetics as observed for interaction with *Tco*ALD^WT^. In the case of *Tco*ALD^L106Y^ and *Tco*ALD^A77E/L106Y^, investigation of the homologous sandwich ELISA format with SPR reveals that non-immobilized Nb474 outcompetes immobilized Nb474 for antigen binding and thus washes the antigen off the Nb474-coated surface. Although the affinity constants for the Nb474-*Tco*ALD^L106Y^ and Nb474-*Tco*ALD^A77E/L106Y^ could not be measured directly, these observations suggest that these mutations weaken the Nb-antigen interaction. For the L106Y mutation, this can again be explained by examination of the structure. The presence of a Tyr residue at this position would disrupt the salt bridge between Asp106 of Nb474 and *Tco*ALD Arg109 and Arg110 (S5B Fig). The A77E/L106Y double mutant most likely experiences a combined effect of both mutations, which is why *Tco*ALD^A77E/L106Y^ yields the lowest binding signals in all experimental set-ups. Together, the SPR and crystallographic data explain the results of the Nb474-based homologous sandwich ELISA. Compared to *Tco*ALD^WT^, a low signal was observed for *Tco*ALD^A77E^, whereas *Tco*ALD^L106Y^ and *Tco*ALD^A77E/L106Y^ could not be detected.

While the Nb474-based immunoassay is highly specific for diagnosing *T*. *congolense* infections, our mutations studies imply that the detection of all *T*. *congolense* strains may not be guaranteed. In our previous work [16], we tested the Nb474-based ELISA on the sera of mice infected with different *T*. *congolense* strains of the Savannah subtype. While some infected sera gave rise to very high signals (*T*. *congolense* strains TC13, IL1180, Ruko 14cl3, and MF3cl2), others displayed low binding (*T*. *congolense* strains STIB68, TRT55, MF5cl4). It is difficult to assess whether these differences arise from i) varying expression levels of *Tco*ALD between the distinct *T*. *congolense* strains, ii) the occurrence of mutations on the epitope recognized by Nb474 with effects similar to the A77E, L106Y, and A77E/L106Y mutations studied in this paper, or iii) a combination of both. Our results concerning the aldolase sequences within the *Nannomonas* subgenus seem to suggest the first hypothesis. The aldolase amino acid sequence conservation among all *T*. *congolense* subtypes tested in this work (Savannah, Forest, Kilifi) is very high (95.2%). Most importantly, the amino acids at positions 77 and 106 are relatively well conserved (Ala77 and Leu106 for Savannah and Kilifi subtypes; Val77 and Leu106 for Forest subtype). While the *T*. *congolense* Forest subtype contains a Val at position 77, this is not expected to severely impact detection in the Nb474-based immunoassay based on our findings. Given that Val and Ala are chemically and structurally much more similar than Glu and Ala, the Nb474-*Tco*ALD interaction is likely to be much less perturbed by the Ala77Val than the Ala77Glu mutation. Hence, this would suggest that the Nb474-based immunoassay would detect all *T*. *congolense* infections. However, the potential occurrence of *T*. *congolense* strains carrying mutations that would escape detection in the Nb474-based ELISA is not unconceivable. This finding calls for an extensive and detailed molecular characterization of the different *T*. *congolense* strains and sequence their genomes. Finally, it is interesting to note that the pig-infective *T*. *simiae* and *T*. *godfreyi* parasites have an aldolase with an Ala77Glu and Leu106 genotype, suggesting that the Nb474-based could be employed to detect infections of these trypanosomes.

**Fig 6:**
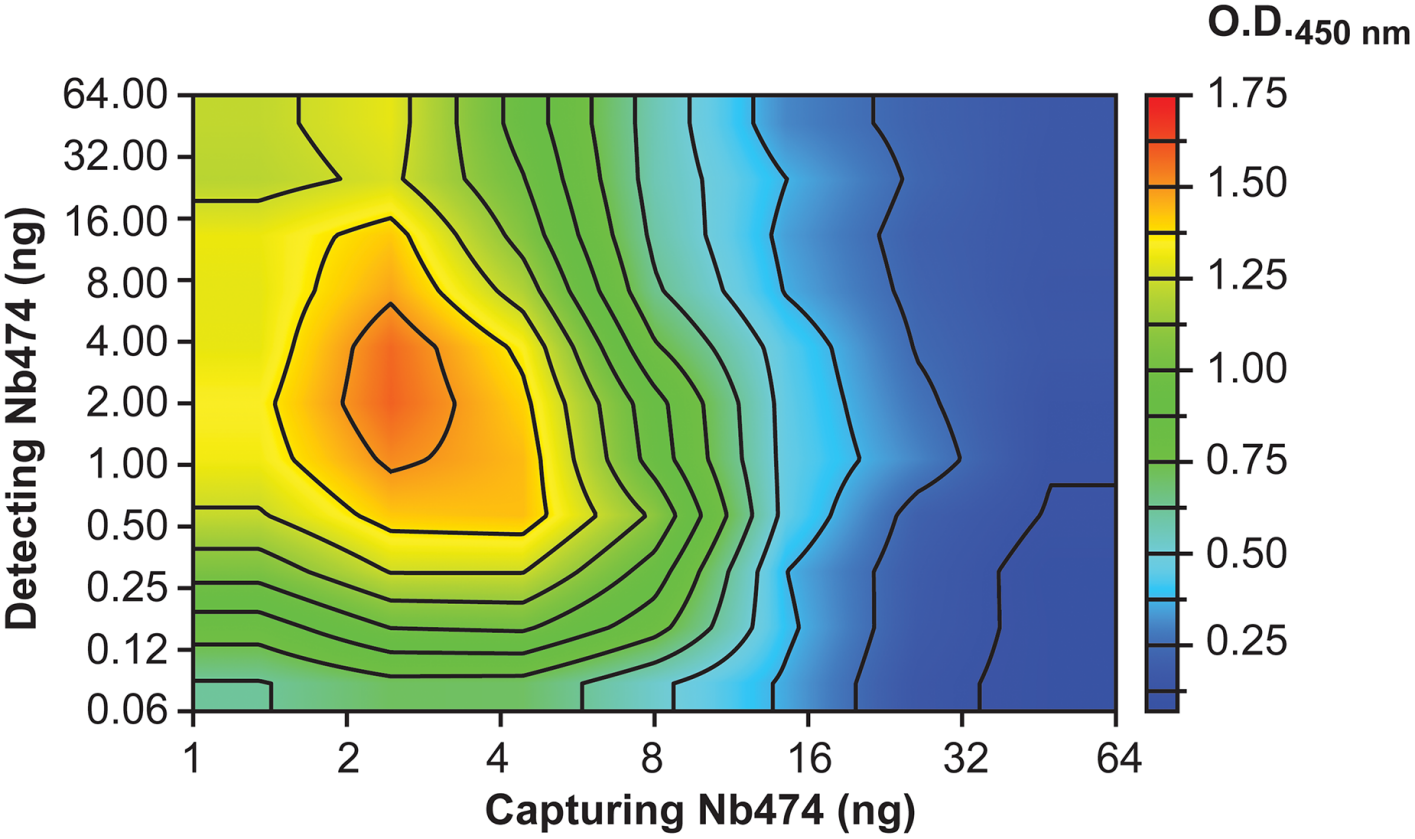
A heat map readily identifies the optimal practical set-up to conduct the Nb474-based homologous immunoassay. Using a checkerboard system, varying amounts of capturing and detecting Nb474 were employed to identify those conditions yielding the highest ELISA signal. The details of this approach are described in [16].

The data presented here also provide insights into the practical set-up of the Nb474-based ELISA. The amounts of capturing and detecting Nb474 yielding the highest signal were determined using a checkerboard ELISA format without prior knowledge of the Nb474-target interaction and its affinity [16]. The outcome of this effort is shown as a heat map in Fig 6. The highest ELISA signal is obtained when relatively low amounts of both capturing and detecting Nb474 are used (~ 2 ng for both, respectively). In the case where the optimal amount of capturing Nb474 is kept fixed (~ 2 ng), any deviation (higher or lower) from the optimal 2 ng amount of detecting Nb474 reduces the intensity of the observed ELISA signal. A decrease results in less detecting Nb474 binding to the Nb474-*Tco*ALD sandwich, which is why a reduction in signal intensity is observed. Based on the results presented in this paper, an increase in the amount of detecting Nb474 above the optimal 2 ng would enhance the “self-competition” or “washing” effect, which was exacerbated in the case of the *Tco*ALD^L106Y^ and *Tco*ALD^A77E/L106Y^ mutants. Likewise, in the case where the optimal amount of detecting Nb474 is kept fixed (~ 2 ng), any deviation from the optimal 2 ng of capturing Nb474 reduces the signal intensity in the ELISA. Employing relatively low amounts of capturing Nb474 is possible due to the high affinity and slow dissociation kinetics of the Nb474-*Tco*ALD interaction as evidenced by the SPR data. A decrease in the amount of capturing Nb474 compared to the optimal case leads to less antigen being captured, which is why a reduction in signal intensity is observed. An increase in the amount of capturing Nb474 would enhance the avidity effects in the ELISA wells, whereby one *Tco*ALD multimer would be able to bind several Nbs simultaneously. Hence, no *Tco*ALD epitopes would be available for binding of detecting Nb474, which results in the observed lower ELISA signal with increasing amounts of capturing Nb474 (Fig 6).

The main focus of this paper was to determine the molecular mechanisms underlying the high specificity of a Nb-based homologous sandwich ELISA that allows detection of *T*. *congolense* infections. As reported previously, the assay targets glycosomal aldolase [16]. While aldolase proteins are relatively well conserved throughout all domains of life, they seem to be immunologically sufficiently distinct, even within the same genus. In this and previous work [16], we have demonstrated that the Nb474-based sandwich assay specifically detects the presence of *T*. *congolense* aldolase, while this is not so for aldolase from other trypanosomes. Coincidentally, a similar finding has been documented for the differential diagnosis of *Plasmodium* infections. A monoclonal antibody-based immunoassay targeting malarial aldolase results in the specific detection of *Plasmodium vivax* infections, while remaining negative for samples containing *Plasmodium falciparum* [43]. These examples demonstrate that parasite-encoded aldolases are suitable biomarkers for the stringent detection of parasite infections. This may present an interesting research avenue for the development of immunoassays for the specific detection of other pathogens. The results presented in this paper indicate that, while the concept and use of such assays are relatively simple, their underlying biochemistry can be quite complex. This may be of particular interest to those developing similar assays.

## Supporting information

S1 Fig Sequence alignment of trypanosomatid aldolase sequences. The colored bars above the sequence alignment represent the percentage of sequence identity: green (100%), green-brown (between 30% and 100%), and red (below 30%).
(TIF)

S2 Fig Overview of the production and purification of *Tco*ALD, optimization of its purification conditions, and co-crystallization with Nb474. (A.) Recombinant production of *Tco*ALD in *E*. *coli* BL21(DE3). Samples of the bacterial culture were taken before induction (*Lane ‘0h’*) and after overnight incubation after induction of gene expression (*Lane ‘18h’*) and analysed by SDS-PAGE (left) and Western blot (right). The band corresponding to His-tagged *Tco*ALD is indicated by the black arrow (MM = 42.6 kDa). *Lane M*, Prestained Protein Molecular Weight Marker (Fermentas). (B.-C.) Purification of *Tco*ALD by IMAC (B.) followed by gel filtration (C.). The inset in panel (C.) shows an SDS-PAGE (top) and Western blot (bottom) analysis of the fractions collected during SEC for the peak indicated by the asterisk ‘*’. (D.) Results of the Thermofluor analysis conducted with *Tco*ALD in an effort to improve its stability during purification. The thermograms recorded in the initial and final SEC purification buffers are indicated in red and pink, respectively. (E.) Crystals of the (Nb474-*Tco*ALD)_4_ complex obtained in 100 mM sodium cacodylate pH 6.5, 200 mM magnesium acetate, 10% PEG 8000 after approximately 14 days at 20°C. (F.) Diffraction pattern of one of the crystals shown in panel (E.). It shows spots up to 2.9 Å (resolution limits are indicated by the grey circles). The dashed box displays an enlargement of the diffraction pattern close to the 2.9 Å resolution limit.
(TIF)

S3 Fig Detailed view of the interactions between Nb474 and *Tco*ALD. Stereo view of the interactions between Nb474 and *Tco*ALD in two orientations (the bottom view is rotated 180° relative to the top view). Nb474 is depicted in cartoon representation and colors are as in Fig 2. For reasons of clarity only the *Tco*ALD residues involved in Nb474 binding are shown and colored in orange. All interacting residues are shown in a stick representation and are indicated by the colored labels. The dashed lines indicate hydrogen bonds or salt bridges (distance values between 2.32 Å and 4.65 Å, also see S1 Table).
(TIF)

S4 Fig Sequence alignment of *Trypanosoma* aldolase sequences. The colored bars above the sequence alignment represent the percentage of sequence identity: green (100%), green-brown (between 30% and 100%), and red (below 30%). The *Trypanosoma* subgenera are indicated.
(TIF)

S5 Fig Binding behavior of the *Tco*ALD mutants based on the structure of the (Nb474-*Tco*ALD)_4_ complex. (A.) Structural basis for the binding behavior of the *Tco*ALD^A77E^ mutant. Nb474 is shown in surface representation and the color code is the same as in Fig 2. Residues Ala77 and Glu77 of *Tco*ALD (orange) and *Tb*ALD (cyan), respectively, are shown in stick representation. (B.) Structural basis for the binding behavior of the *Tco*ALD^L106Y^ mutant. Nb474 is shown in cartoon representation and the color code is the same as in Fig 2. The residues of *Tco*ALD, and *Tb*ALD are shown in stick representation and colored as in (A.).
(TIF)

S1 Table List of interactions between Nb474 and *Tco*ALD. Distances only given in case of hydrogen bonds or electrostatic interaction. Also see S3 Fig.
(DOCX)
