# Supplementary figures and images for: Structural basis for the high specificity of a Trypanosoma congolense immunoassay targeting glycosomal aldolase
Source: PLoS Negl Trop Dis. 2017 Sep 15;11(9):e0005932. doi: 10.1371/journal.pntd.0005932 (PMC5617235; doi:10.1371/journal.pntd.0005932)

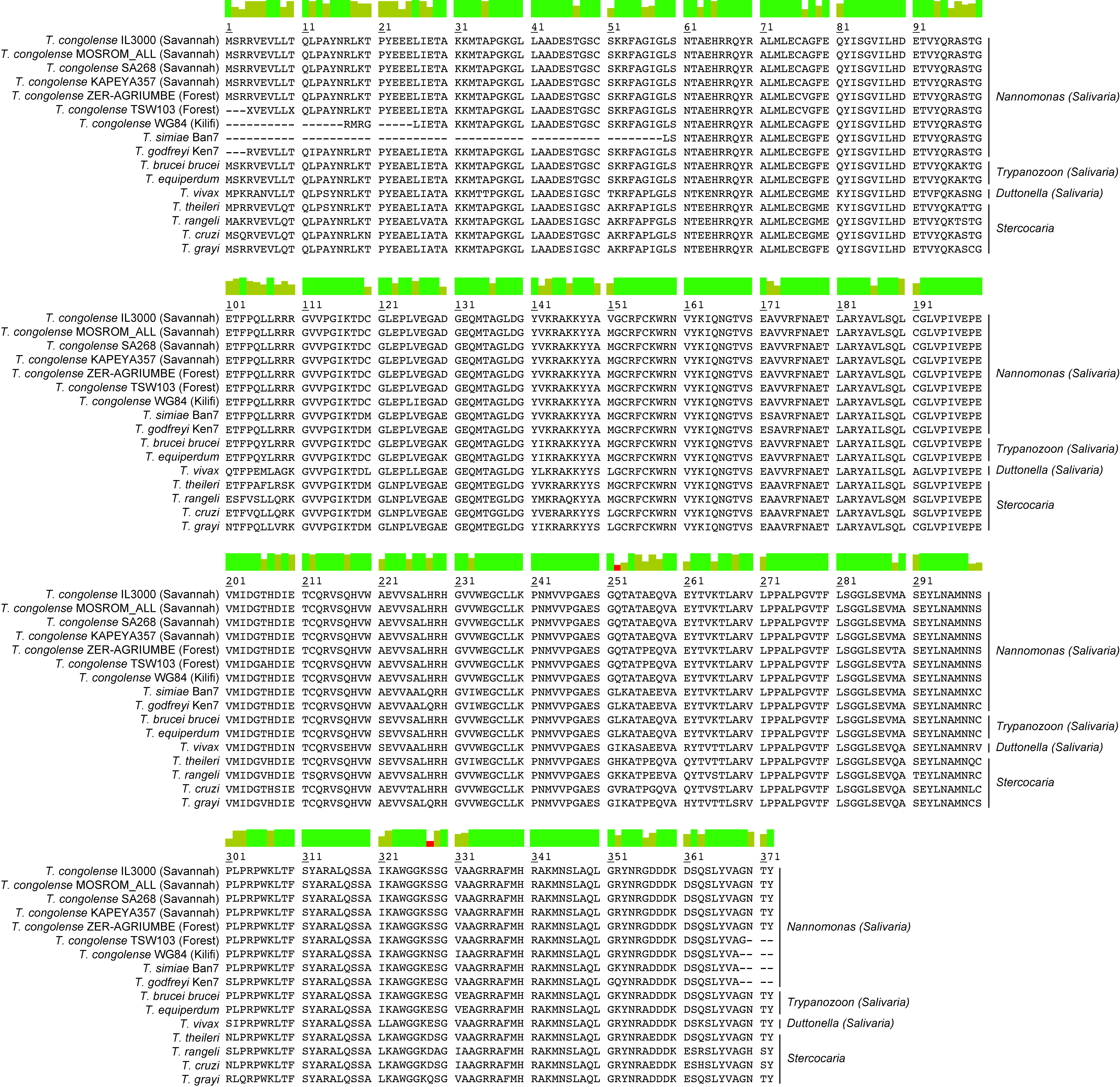

Supplement: S4 Fig — The colored bars above the sequence alignment represent the percentage of sequence identity: green (100%), green-brown (between 30% and 100%), and red (below 30%). The Trypanosoma subgenera are indicated. (TIF) [file pntd.0005932.s004.tif]

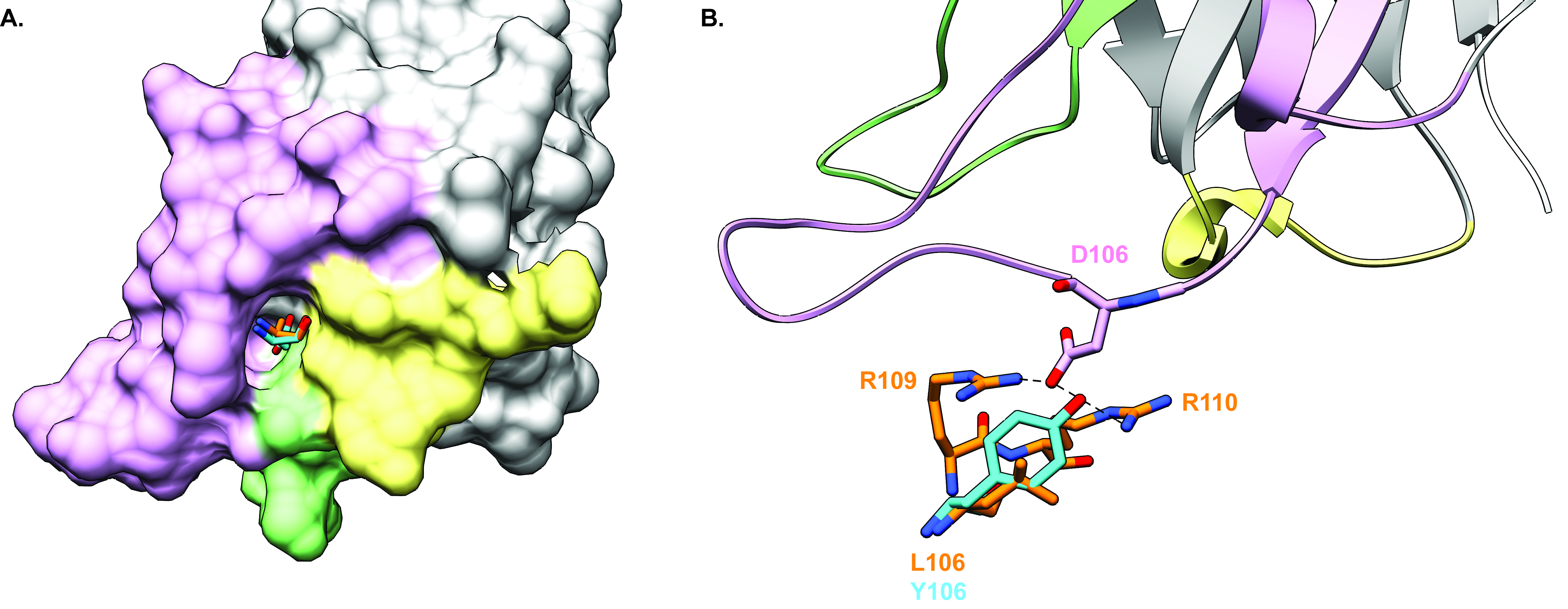

Supplement: S5 Fig — (A.) Structural basis for the binding behavior of the TcoALDA77E mutant. Nb474 is shown in surface representation and the color code is the same as in Fig 2. Residues Ala77 and Glu77 of TcoALD (orange) and TbALD (cyan), respectively, are shown in stick representation. (B.) Structural basis for the binding behavior of the TcoALDL106Y mutant. Nb474 is shown in cartoon representation and the color code is the same as in Fig 2. The residues of TcoALD, and TbALD are shown in stick representation and colored as in (A.). (TIF) [file pntd.0005932.s005.tif]
