# Supplementary material for: Structural basis for the high specificity of a Trypanosoma congolense immunoassay targeting glycosomal aldolase
Source: PLoS Negl Trop Dis. 2017 Sep 15;11(9):e0005932. doi: 10.1371/journal.pntd.0005932 (PMC5617235; doi:10.1371/journal.pntd.0005932)
Supplement: S1 Table — Distances only given in case of hydrogen bonds or electrostatic interaction. Also see S3 Fig. (DOCX) [file pntd.0005932.s006.docx]

**S1 Table. List of interactions between Nb474 and *Tco*ALD.** Distances only given in case of hydrogen bonds or electrostatic interaction. Also see S3 Fig.

| Nb474 |  |  | *Tco*ALD |  |  |
| --- | --- | --- | --- | --- | --- |
| Residue | **Group** | **FR/CDR** | **Residue** | **Group** | **Interaction (distance)** |
| Val2 | side chain | FR | Lys343 | side chain | Van der Waals |
| Glu26 | side chain | CDR1 | Lys343 | side chain | electrostatic  (3.92 Å) |
| Ala28 | backbone NH | CDR1 | Gln81 | side chain CO | hydrogen bond  (3.25 Å) |
| Tyr31 | side chain | CDR1 | Gln81 | side chain | Van der Waals |
| Tyr31 | side chain | CDR1 | Glu80 | side chain | Van der Waals |
| Tyr31 | side chain | CDR1 | Gly78 | side chain | Van der Waals |
| Tyr32 | side chain | CDR1 | Gln81 | side chain NH | hydrogen bond  (3.11 Å) |
| Tyr32 | side chain | CDR1 | Ala77 | backbone CO | hydrogen bond  (3.90 Å) |
| Tyr32 | side chain | CDR1 | Gly78 | side chain | Van der Waals |
| Arg53 | side chain | CDR2 | Glu80 | side chain | electrostatic  (3.70 Å) |
| Thr104 | backbone CO | CDR3 | Ala77 | backbone NH | hydrogen bond  (2.91 Å) |
| Thr105 | side chain | CDR3 | Arg335 | side chain | hydrogen bond  (3.31 Å) |
| Asp106 | backbone NH | CDR3 | Glu75 | backbone CO | hydrogen bond  (2.96 Å) |
| Asp106 | side chain | CDR3 | Arg109 | side chain | electrostatic  (2.32 Å) |
| Asp106 | side chain | CDR3 | Arg110 | side chain | electrostatic  (3.47 Å) |
| Tyr108 | side chain | CDR3 | Arg109 | side chain | Van der Waals |
| Tyr108 | side chain | CDR3 | Glu101 | side chain | Van der Waals |
| Ser110 | backbone NH | CDR3 | Thr99 | backbone CO | hydrogen bond  (3.04 Å) |
| Ser110 | side chain | CDR3 | Thr99 | backbone CO | hydrogen bond  (2.66 Å) |
| Ser110 | side chain | CDR3 | Gly100 | backbone CO | hydrogen bond  (3.79 Å) |
| Asp125 | side chain | CDR3 | Arg336 | side chain | electrostatic  (2.59 Å) |
| Tyr126 | side chain | CDR3 | Arg336 | backbone CO | hydrogen bond  (4.53 Å) |
| Tyr126 | side chain | CDR3 | Arg335 | backbone CO | hydrogen bond  (4.65 Å) |
